# Supplementary material for: Identification and Functional Analysis of Tomato CIPK Gene Family
Source: Int J Mol Sci. 2019 Dec 23;21(1):110. doi: 10.3390/ijms21010110 (PMC6981861; doi:10.3390/ijms21010110)
Supplement: Supplementary file 1 [file ijms-21-00110-s001.pdf]

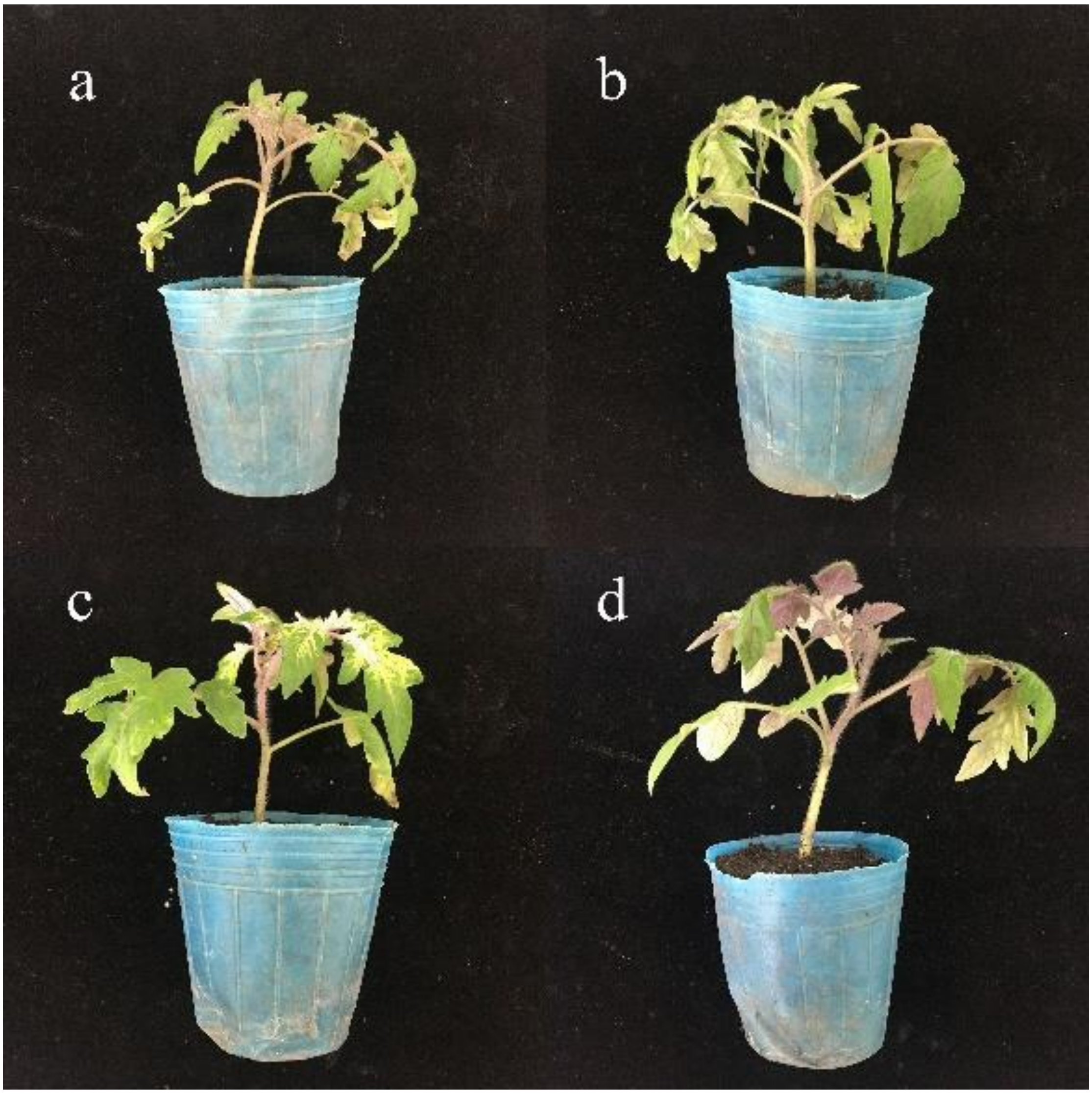

Supplement Figure 1. Observation of plant symptoms after virus induced gene silencing (VIGS). **a:** SlCIPK1 silenced plant; **b**: SlCIPK8 silenced plant; **c**: PDS silenced plant; **d**: control.

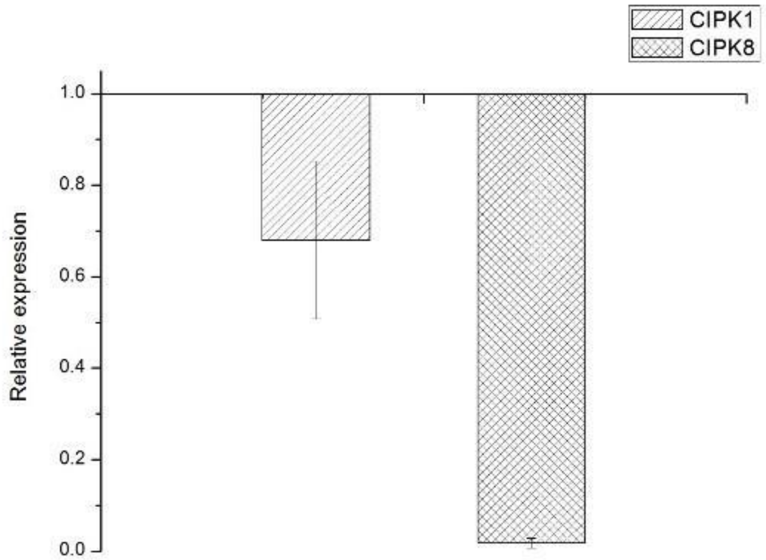

Supplement Figure 2. Detection of gene expression after virus induced gene silencing (VIGS).

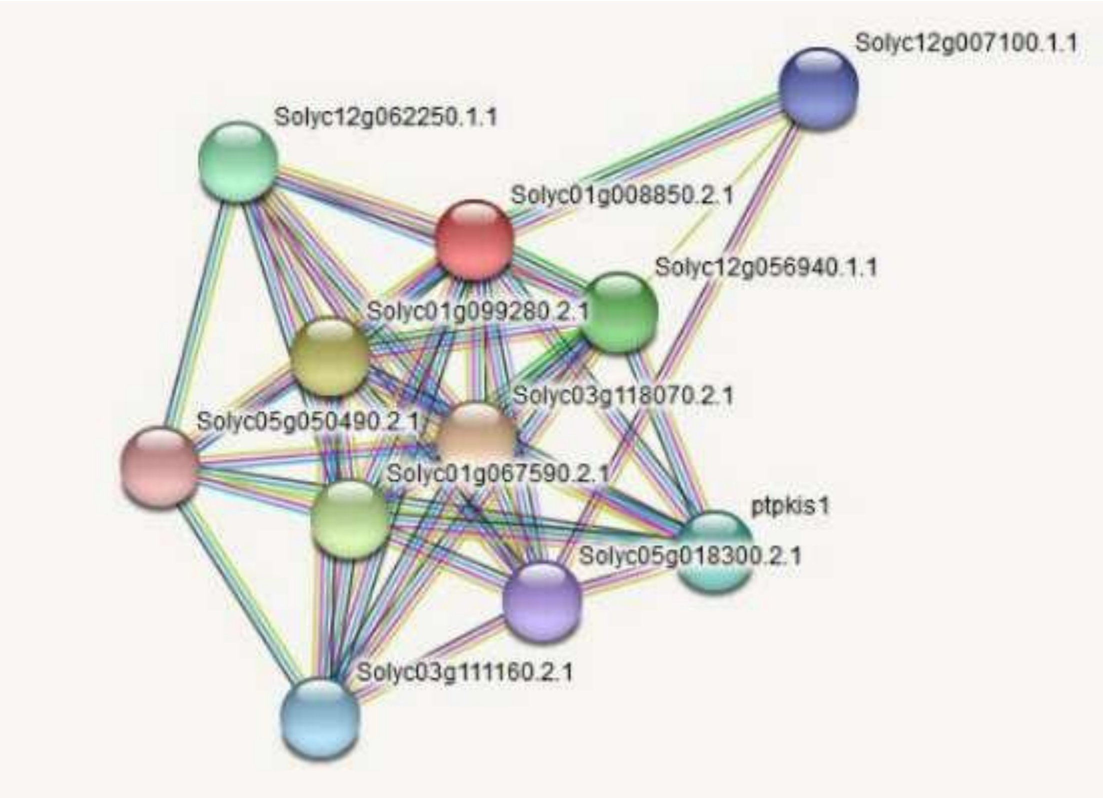

Supplement figure 3. Prediction of protein network interactivity of SlCIPK1 protein.

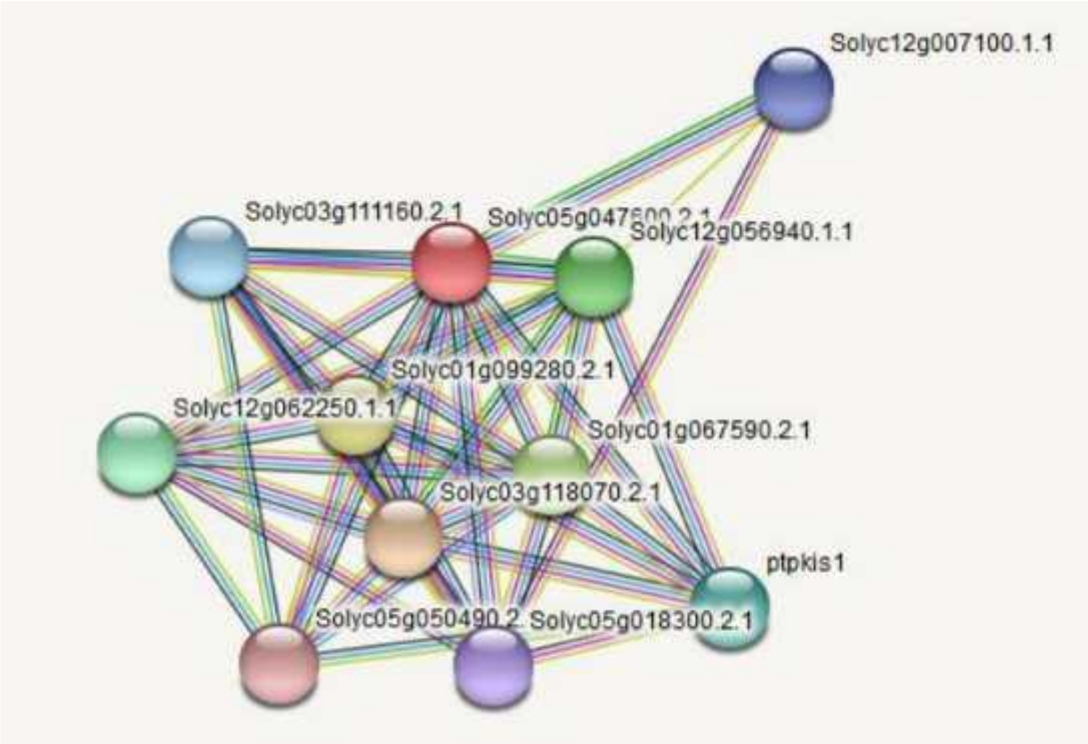

Supplement figure 4. Prediction of protein network interactivity of SlCIPK8 protein.

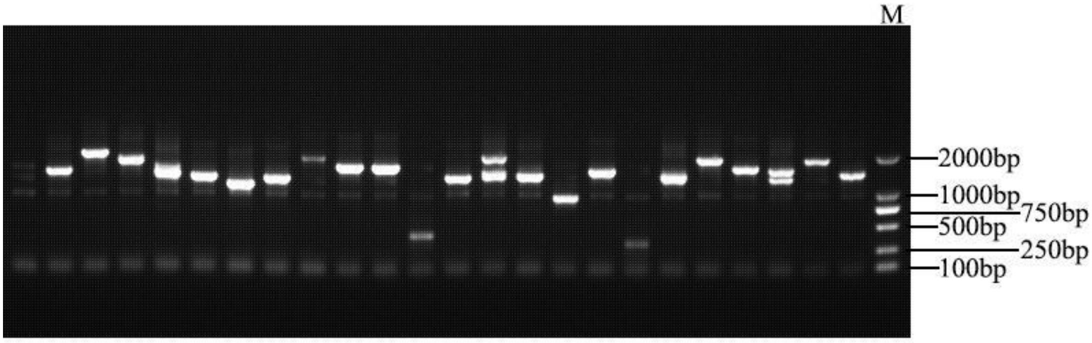

Supplement figure 5. Recombination rate of the yeast library insert fragment.
